# Interventional Management of Intracardiac Masses

**DOI:** 10.1016/j.jscai.2026.104401

**Published:** 2026-03-12

**Authors:** Pedro Engel Gonzalez, James A. Goldstein

**Affiliations:** a Division of Cardiology, Department of Medicine, Henry Ford Health, Detroit, Michigan; b Department of Cardiovascular Medicine, Beaumont University Hospital, Corewell Health, Royal Oak, Michigan

**Keywords:** cerebral embolic protection, intracardiac masses, mitral valve

Intracardiac masses (thrombi, vegetations, and tumors) threaten potentially catastrophic embolic events. Interventional techniques based on transcatheter aspiration systems now provide nonsurgical options for extirpation of many such masses. These US Food and Drug Administration-approved techniques have increasingly been employed for debulking right-sided intracardiac masses, with successful relief of thrombi in transit, vegetations on the tricuspid valve and electrophysiologic leads, and even tumors, thereby reducing the risk of problematic pulmonary embolic events.

Left-sided intracardiac masses pose far greater clinical risk attributable to the potentially catastrophic nature of systemic cardioembolic events, particularly major ischemic stroke. The standard of care for left heart masses has traditionally consisted of “watchful” medical management when feasible, with surgical intervention when patients have failed medical therapy. Catheter-based techniques for aspiration of left heart masses are performed when the heart team deems that the patient is not a surgical candidate, often due to age and a high burden of comorbidities, and when medical therapy has not been sufficient. These techniques are also inhibited by risk of fragmentation leading to stroke.

In this issue of *JSCAI*, Nandhakumar and colleagues^1^ present a case report demonstrating the feasibility of percutaneous snare extraction of a left-sided mitral valvular vegetation, employing “near-total-body embolic protection” by deployment of 2 SENTINEL cerebral embolic protection devices (Boston Scientific) (one via the right radial artery to protect the right and left internal carotid arteries and a second device positioned to protect the vertebral artery with the proximal filter partially covering the descending aorta). The authors should be congratulated for this novel approach, which facilitated successful and safe extraction of the vegetation. Given that the SENTINEL filters were designed for carotid diameters, it is not surprising that debris embolized to the popliteal trifurcation requiring surgical embolectomy, a “small price to pay” for pre-empting a recurrent stroke.

Whereas catheter-based intervention of right heart masses may result in fragmentation and resultant pulmonary emboli, the robust nature of the pulmonary circulation renders it tolerant to small emboli, and the same interventional techniques are readily applied to large emboli of the pulmonary arteries. In contrast, in the left heart, fragmentation debris from friable vegetations or thrombi resulting in even small emboli may be catastrophic. The feasibility of this technique has been previously reported with a good safety profile in a single-center case series. In these cases, the use of the SENTINEL device was via a biradial approach in which there was “near-total brain protection” with the filters offering protection to the right and left internal carotid arteries, and an additional filter protecting the vertebral artery.^2^^,^^3^ There are ongoing efforts to accumulate more evidence in this space.

The present case documents the feasibility of catheter-based management of selected left heart cardiac masses. The major innovation described is the strategy of dual systemic arterial embolic protection. Although embolic protection was not complete in the present case, it is reasonable to speculate that newer systems designed to achieve “comprehensive arterial protection” will become available. It should be noted that other approaches may prove attractive and augment the portfolio of management options. Prior reports have described transient deployment of an undeployed WATCHMAN device (Boston Scientific) in the ascending aorta to provide systemic embolic protection during left atrial appendage closure in the presence of thrombus,^4^ as well as the use of the ŌNŌ retrieval device as a conceptual global embolic “safety net.”^5^

Transcatheter extracorporeal membrane oxygenation-assisted and mechanical vacuum-assisted extraction in the systemic circulation have also been reported.^6^ Further clinical data are necessary to delineate the efficacy of these various management options.^7^ Clearly, for such complex patients, a team-based approach will yield the greatest value for individual decision making.

Finally, it should be noted that the present case employing off-label adaptation of currently available tools emphasizes the innovative nature inherent to our field. Such early proof-of-concept steps serve as both the stimulus and bedrock that has characterized the transformation in interventional cardiovascular care these past 3 decades.

## Peer review statement

Associate Editor James A. Goldstein had no involvement in the peer review of this article and has no access to information regarding its peer review. Full responsibility for the editorial process for this article was delegated to Editor-in-Chief Alexandra J. Lansky.

## Declaration of competing interest

Pedro Engel Gonzalez serves as a consultant and proctor for Edwards Lifesciences. James A. Goldstein reported no financial interests.
